# Methodological Considerations in Assessing HALP Score as a Prognostic Marker in Rheumatic Mitral Stenosis

**DOI:** 10.1002/clc.70326

**Published:** 2026-04-20

**Authors:** Muhammad Talha, Syed Huzaifa Khan, Ata ullah

**Affiliations:** ^1^ Nowshera Medical College Nowshera Pakistan

Dear Editor,

We read with genuine interest the recent article examining the prognostic role of the HALP score in rheumatic mitral stenosis [1]. The topic is clinically relevant, and the effort to identify accessible risk markers is commendable. However, a few methodological aspects merit closer consideration before firm conclusions can be drawn.

One key concern relates to the composition of the primary endpoint. The inclusion of physician‐driven mitral valve interventions alongside outcomes such as death and stroke introduces an important source of bias. Unlike hard endpoints, interventions are influenced by clinical judgment and practice patterns. Patients with lower HALP scores may be perceived as higher risk, prompting earlier intervention, while those with higher scores might be managed more conservatively. In this context, the composite endpoint risks reflecting decision‐making behavior rather than true disease progression. Notably, the study does not present an analysis restricted to hard outcomes alone, making it difficult to determine whether HALP independently predicts events such as death or stroke. As highlighted in prior methodological work, combining hard and soft endpoints can obscure interpretation, particularly when the softer components drive the results [2].

A second issue is statistical power. With a relatively small number of outcome events and several covariates included in the Cox model, the analysis likely falls short of recommended event‐to‐variable thresholds. This raises concerns about model stability and interpretability. The borderline association observed for HALP may therefore reflect limited power rather than a definitive absence (or presence) of effect. In its current form, the analysis does not allow a confident assessment of HALP as an independent predictor [3].

Taken together, these considerations suggest that while HALP remains a promising and biologically plausible marker, the present findings should be interpreted with caution. A re‐analysis focusing on hard clinical endpoints and a more parsimonious modeling approach would help clarify its true prognostic value.

We appreciate the authors' contribution to this evolving area and hope these comments are received in the constructive spirit intended.

## Author Contributions

All authors have read and approved the final manuscript.

## Funding

The authors have nothing to report.

## Ethics Statement

The authors have nothing to report.

## 1 Conflicts of Interest

The authors declare no conflicts of interest.

## Data Availability

The authors have nothing to report.
